# The clinical and immunological features of the post-extracorporeal shock wave lithotripsy anti-glomerular basement membrane disease

**DOI:** 10.1080/0886022X.2020.1869042

**Published:** 2021-01-12

**Authors:** Beining Wang, Jia Xiaoyu, Xiaojuan Yu, Zhao Cui, Minghui Zhao

**Affiliations:** a Renal Division, Peking University First Hospital, Beijing, China; b Institute of Nephrology, Peking University, Beijing, China; c Key Laboratory of Renal Disease, Ministry of Health of China, Beijing, China; d Key Laboratory of CKD Prevention and Treatment, Ministry of Education of China, Beijing, China; e Research Units of Diagnosis and Treatment of Immune-mediated Kidney Diseases, Chinese Academy of Medical Sciences, Beijing, China; f Peking-Tsinghua Center for Life Sciences, Beijing, China

**Keywords:** ESWL, lithotripsy, anti-glomerular basement membrane disease, antigen

## Abstract

**Introduction:**

Extracorporeal shock wave lithotripsy (ESWL) is a noninvasive modality to treat urolithiasis, with complications including tissue damage and hematoma of kidney parenchyma. Anti-glomerular basement membrane (GBM) disease is suggested to be a rare complication of ESWL since it was reported in several cases to occur after ESWL. However, the clinical and immunological features of the ESWL-associated anti-GBM disease have not been fully investigated so far.

**Case Presentation:**

Here, we present the clinical, pathological, and immunological characteristics of three patients with the post-ESWL anti-GBM disease in our hospital. Anti-GBM disease occurred within a median of 22 months after ESWL treatment. It presented with similar clinical features to the classic anti-GBM disease, including fever, gross hematuria, and rapidly progressive glomerulonephritis (RPGN) with poor renal prognosis. Sera from all patients recognized the α3(IV)NC1 in GBM, but with IgG2 and IgG4 as the dominant IgG subclasses.

**Conclusion:**

Although further exploration is required to prove the causal relationship in this rare condition, our study reminds physicians that patients developing acute renal insufficiency after ESWL should lead to the suspicion of anti-GBM disease and in-time diagnosis and treatment.

## Introduction

Anti-glomerular basement membrane (GBM) disease or Goodpasture (GP) disease is a rare but fulminant and fatal disorder. It is widely accepted as a prototypical autoimmune disease induced by autoantibodies targeting the glomerular and alveolar basement membrane [1,2]. The well-defined autoantigen is localized to the non-collagen domain (NC1) of the α3 chain of type IV collagen [α3(IV)NC1] in GBM [3]. In addition, the autoantibodies have been proven to recognize the other 4 α chains (α1, 2, 4, and 5) within type IV collagen [4,5]. Two conformational epitopes, EA and EB, were mapped inside α3(IV)NC1 [6] and sequestered within the GBM in healthy individuals [7]. It is still unknown how the immune tolerance would be breached and epitopes exposed. One of the accepted mechanisms is that factors such as smoking [8], hydrocarbon exposure [9], infection [10], or preceding glomerular diseases [11–13] would damage the basement membrane and unmask the epitopes [14]. Rare cases have been reported about anti-GBM diseases associated with urinary obstruction [15–18] or nephrectomy [19].

Extracorporeal shock wave lithotripsy (ESWL) is a common and noninvasive modality to treat urolithiasis, disintegrating stones by shock waves. Although generally safe [20], it could cause short-term complications as renal colic or obstruction by stone fragments, tissue damage or hematoma, and even temporary reduction of glomerular filtration rate [21]. Renal histopathological findings of human and animal models shortly after ESWL included focal damage to vascular endothelium, nephron, renal tubules, and interstitia [20,22–24]. In 1990, Guerin et al. first reported a 67-year-old male with rapidly progressive renal disfunction 7 months after ESWL. His serum anti-GBM antibody was positive, while the serum before ESWL was not [25]. Since then, there have been several case reports about the post-ESWL anti-GBM disease, proposing anti-GBM disease as a rare complication of ESWL [26–29]. However, the causal relationship between the two has not been elucidated yet, nor the antigen spectrum or IgG subclasses of the autoantibodies in this rare entity.

In this study, we identified three patients with the post-ESWL anti-GBM disease after retrospectively reviewing 166 consecutive patients diagnosed with the anti-GBM disease in Peking University First Hospital from January 1, 2010 to January 31, 2020. We analyzed their clinical, pathological, and immunological features and reviewed five previously reported cases, aiming to draw a more comprehensive picture and bring insight into possible etiologies of this rare condition.

## Case report

### Case 1

A 36-year-old male was otherwise healthy except for undergoing one session of ESWL for kidney stones 24 months ago. Kidney function (represented as serum creatinine) was normal. Twenty-four months later, he presented with fever, cough, and hemoptysis for 5 days. He was anemic with hemoglobin 75 g/L and serum creatinine was 691 μmol/L (eGFR, 8 mL/min/1.73m^2^). Two days later, he developed gross hematuria and serum creatinine rose to 884 μmol/L (eGFR, 6 mL/min/1.73m^2^), at which time he was referred to our hospital. He was a long-term cigarette smoker and had gasoline exposure 15 days ago.

On admission, he was pale and febrile with no lymphadenopathy. Breath sounds were weak in both lungs on auscultation. Serum creatinine rapidly increased to 1279 μmol/L (eGFR, 4 mL/min/1.73m^2^) with oliguria and hemoglobin decreased to 49 g/L. Chest radiography suggested bilateral pulmonary hemorrhage. The circulating anti-GBM antibody was positive, with the titer >200 RU/mL (Euroimmune ELISA kit, normal range <20 RU/mL), while the serum anti-neutrophil cytoplasmic antibody (ANCA) was undetected (Table 1).

**Table 1.** Clinical, pathological, and immunological features of patients with post-ESWL anti-GBM disease in our hospital.

	Patient 1	Patient 2	Patient 3
Sex/age, y	M/36	M/50	F/74
Hydrocarbon exposure (Y/N)	Y	N	N
Smoking (Y/N)	Y	Y	N
Prodromal infection (Y/N)	Y	N	Y
Fever (Y/N)	Y	Y	Y
Pulmonary hemorrhage (Y/N)	Y	N	N
Gross hematuria (Y/N)	Y	Y	Y
Oliguria/Anuria (Y/N)	Y	N	Y
Hemoglobin on diagnosis, g/L	49.0	102.0	63.0
Serum albumin, g/L	30.0	27.1	29.0
Urinary protein, g/24h	2.0	1.6	0.6
Nephrotic syndrome (Y/N)	N	N	N
Serum creatinine on diagnosis, μmol/L	1279	364	1066
eGFR on diagnosis, mL/min/1.73m^2^	4.0	15.0	3.0
Anti-GBM antibodies on diagnosis, RU/mL	>200	183	196
Positive ANCA (Y/N)	N	N	N
Treatment	PE/MP/Pred/CTX	PE/MP/Pred	MP/Pred/CTX
Anti-GBM antibody on discharge, RU/mL	33	51	102
Serum creatinine on discharge, μmol/L	425	353	618
eGFR on discharge, mL/min/1.73 m^2^	14.0	16.3	5.3
Dialysis-dependent on discharge (Y/N)	N	Y	Y
Duration of follow-up, m	3	60	48
Dialysis-dependent at last follow-up (Y/N)	N	Y (PD)	Y (HD)
Death at last follow-up (Y/N) (cause of death)	N	N	Y (Esophagus cancer)
Stone location	kidney	ureter	kidney
Total ESWL number	1	1	1
ESWL-to-onset interval, m	24	22	10
Anti-α1(IV)NC1 antibody (ref range, <0.04)	0.06	0.89	0.33
Anti-α2(IV)NC1 antibody (ref range, <0.05)	0.01	0.83	0.12
Anti-α3(IV)NC1 antibody (ref range, <0.06)	2.11	2.08	2.15
Anti-α4(IV)NC1 antibody (ref range, <0.58)	0.27	1.43	0.70
Anti-α5(IV)NC1 antibody (ref range, <0.02)	0.00	0.48	0.10
Anti-α3EA antibody (ref range, <0.07)	1.39	1.67	1.61
Anti-α3EB antibody (ref range, <0.15)	0.59	1.54	1.35
Anti-α3(IV)NC1 IgG1 (ref range, <0.02)	0.68	0.57	0.73
Anti-α3(IV)NC1 IgG2 (ref range, <0.07)	1.51	1.32	1.25
Anti-α3(IV)NC1 IgG3 (ref range, <0.01)	0.53	0.49	0.63
Anti-α3(IV)NC1 IgG4 (ref range, <0.01)	2.34	0.56	0.43

M: male; F: female; eGFR: estimated glomerular filtration rate; GBM: glomerular basement membrane; ANCA: anti-neutrophil cytoplasmic antibody; PE: plasma exchange; MP: methylprednisolone pulse; Pred: prednisone; CTX: cyclophosphamide; PD: peritoneal dialysis; HD: hemodialysis; ESWL: extracorporeal shock wave lithotripsy; IgG: immunoglobulin G; ref: reference; Y: yes; N: no.

Anti-GBM disease was diagnosed based on the renal-pulmonary involvement and positive serum anti-GBM antibody. The patient was prompted to plasma exchange, intravenous methylprednisolone pulse treatment, cyclophosphamide, and hemodialysis. After 17 sessions of plasma exchange, he became afebrile without hemoptysis. His serum anti-GBM antibody decreased to 33 RU/ml and serum creatine to 425 μmol/L (eGFR, 14 mL/min/1.73m^2^). Repeated chest radiography indicated the absorption of his hemorrhage. He was dialysis-independent on discharge and a 3-month follow-up.

Sera from this patient were tested for antigen spectrum using recombinant human α1–5(IV)NC1 chains and chimeric proteins containing epitopes EA and EB on α3(IV)NC1 (Supplementary materials and methods). The patient's serum recognized α1 and α3(IV)NC1 domains, and both epitopes EA and EB of α3(IV)NC1. The immunoglobulin G (IgG) subclass distribution against α3(IV)NC1 was also tested for the sera. All 4 subclasses of IgG were detected, with the dominance of IgG2 and IgG4 (Table 1; Figure S1).

**Figure 1.**
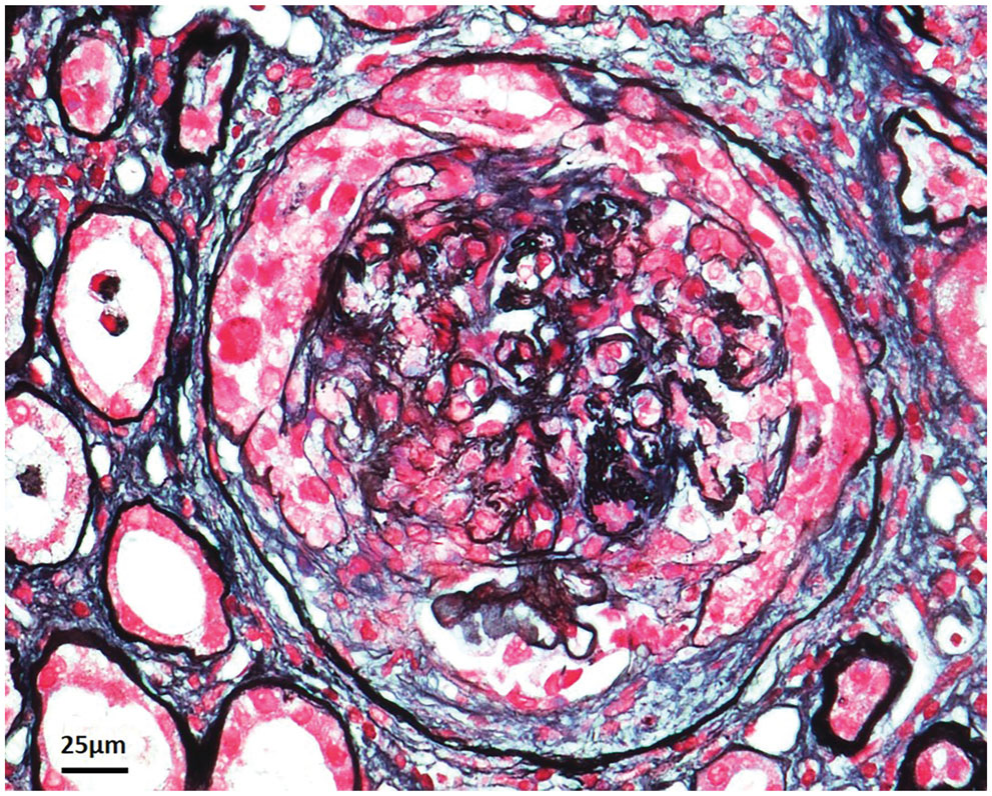
Renal pathology of patient 3 with post-extracorporeal shock wave lithotripsy (ESWL) anti-GBM disease showed cellular crescent formation in a glomerulus by periodic acid-silver methenamine and Masson trichrome stain on light microscopy (400×).

### Case 2

A 50-year-old male undertook one session of ESWL for ureter stones with normal kidney function 22 months ago. He was a 20-year cigarette smoker but had quit smoking for 10 years. Twenty-two months later he was referred to our hospital for fever, gross hematuria, and rapidly progressive renal dysfunction. He had suffered from intermittent fever for 2 months without other indicating symptoms and it could not be resolved by antibiotics. Fifteen days prior to admission, he developed gross hematuria and the serum creatinine progressed from 160 μmol/L (eGFR, 40 mL/min/1.73m^2^) to 504 μmol/L (eGFR, 11 mL/min/1.73m^2^). No stones were found by renal ultrasound.

After admission, he was diagnosed with anti-GBM disease due to positive serum anti-GBM antibody (183 RU/mL). His serum ANCA was tested negative. He received 7 sessions of plasma exchange, intravenous methylprednisolone pulse treatment, and hemodialysis. His condition improved and hematuria disappeared afterward. Serum creatinine decreased to 353 μmol/L (eGFR, 16 mL/min/1.73m^2^) and anti-GBM antibody titer to 51 RU/mL on discharge. The patient was dialysis-dependent during a 60-month follow-up (Table 1).

The patient's sera reacted with all five α chains and both EA and EB epitopes of α3(IV)NC1. All 4 IgG subclasses to α3(IV)NC1 were detected with the dominance of IgG2 (Table 1; Figure S1).

### Case 3

A 74-year-old female received one session of ESWL for kidney stones 10 months ago when the renal function was normal. Ten months after ESWL, she complained of fever and gross hematuria with urinary irritation symptoms for 2 weeks. No leukocytosis was found, but her urinalysis revealed white and red blood cells. Initial workups showed elevated C-reactive protein (96 mg/L) and normal serum creatinine (87 μmol/L; eGFR, 55 mL/min/1.73m^2^). Renal ultrasound showed no stones. She was diagnosed with urinary tract infection and treated by intravenous antibiotics, after which she reported no symptom improvement but developed oliguria and bilateral edema of lower extremities. Her serum creatinine deteriorated to 1066 μmol/L (eGFR, 3 mL/min/1.73m^2^) within 9 days. Then she was referred to our hospital.

On admission, physical examination revealed a sick and pale woman with no rales on auscultation. Serum anti-GBM antibody was positive (196 RU/mL) while the serum ANCA was not. Renal biopsy revealed severe necrosis of glomerular capillary walls and cellular/cellular-fibrous crescents in all 32 glomeruli on light microscopy (Figure 1), with no electron-dense deposits on electron microscopy. The immunofluorescence microscopy was negative on a sclerotic glomerulus. She was treated by methylprednisolone pulse and cyclophosphamide. Plasma exchange was not performed, given the poor prognosis based on renal biopsy. Her renal function did not recover and she remained dialysis-dependent. The patient died of esophagus cancer 2 years after discharge.

The patient's sera recognized all five α chains and both EA and EB epitopes of α3(IV)NC1. As for circulating IgG to α3(IV)NC1, all four subclasses were detected with the dominance of IgG2 (Table 1; Figure S1).

## Discussion

In this study, we described three patients with anti-GBM disease occurring within 2 years after ESWL. The incidence of such cases among all anti-GBM patients from our hospital in the past 10 years was 1.81% (3/166). Their clinical manifestations, pathological features, and antigen spectrum were similar to the classic anti-GBM disease. All three patients presented with fever, gross hematuria, and rapidly progressive glomerulonephritis (RPGN) with poor renal prognosis. One of them suffered from pulmonary hemorrhage. The one available renal biopsy revealed 100% crescent formation in the glomeruli. All three patients recognized a3(IV)NC1 and EA/EB epitopes. None were positive for anti-neutrophil cytoplasmic antibody (ANCA).

Post-ESWL anti-GBM disease has been rarely reported. After searching in PubMed using keywords ‘anti-GBM’, ‘Goodpasture’, ‘ESWL’, and ‘lithotripsy’, we found five previous cases of the post-ESWL anti-GBM disease with full text in English, reviewed in Table 2. Among the five patients, three of them were male and two were female, with a median age of 67 years old (range, 32–72 years). Human leukocyte antigen (HLA) phenotyping was performed in all five patients, with four of them expressing susceptible serotypes for anti-GBM disease [30]. All previous cases presented with shorter ESWL-to-onset intervals (1 week to 7 months) than ours, possibly due to more ESWL treatment numbers. Renal damage by ESWL appears to be cumulative in animal experiments and human, proportional to the application frequency and the number of treatments [31–33]. Their clinical manifestations, renal pathologies, and prognosis were similar to ours. Specifically, fever occurred in all previous reports and ours. However, only a few of them (3/8, 37.5%) were reported to bear prodromal infections and none in either group were positive for ANCA. Fever was a common feature in anti-GBM disease patients, as shown in our previous study in a 140-patient cohort, and 78.7% of febrile patients had infections [34]. The infection rate in post-ESWL anti-GBM disease patients seemed to be lower, which might indicate distinct disease triggers other than prodromal infections in these patients.

**Table 2.** Clinical and pathological data of previously reported post-ESWL anti-GBM disease cases.

	1	2	3	4	5
Authors	Cranfield et al.	Sellin et al.	Xenocostas et al.	Iwamoto et al.	Guerin et al.
Publication year	2015	2005	1999	1998	1990
Sex/age, y	F/67	M/32	M/72	F/37	M/67
Hydrocarbon exposure (Y/N)	NA	NA	NA	NA	NA
Smoking (Y/N)	NA	NA	NA	NA	Y
Prodromal infection (Y/N)	N	N	Y	N	N
Fever (Y/N)	Y	Y	Y	Y	Y
Pulmonary hemorrhage(Y/N)	Y	N	N	N	N
Gross hematuria (Y/N)	NA	Y	N	Y	NA
Serum creatinine on diagnosis, μmol/L	1179	919	NA	1114	1074
Positive anti-GBM antibody(Y/N)	Y	Y	Y	Y	Y
Positive ANCA (Y/N)	N	NA	N	N	N
HLA phenotype	DR4, DQ6	DRB1*11 & 13	DR15	DR2	DR2
Treatment	PE/MP/Pred/CTX	Pred/CTX	NA	PE/MP/Pred/CTX	NA
Dialysis-dependent (Y/N)	Y	Y	Y	Y	Y
Renal biopsy (Y/N)	NA	Y	Y	Y	Y
Immunofluorescence	NA	Linear IgG deposits along GBM	Linear IgG deposits along GBM	Linear IgG and C3 deposits along GBM	Linear IgG and C3 deposits along GBM
Light microscopy	NA	Crescent formation in 23/25 glomeruli	Crescent formation in nearly all glomeruli	Cellular crescents in all glomeruli	Cellular crescents in all glomeruli
Electron-dense deposits on electron microscopy	NA	NA	N	N	NA
Stone location	NA	Infundibulum	Left renal pelvis	Right kidney	Left kidney
Stone component	NA	Calcium oxalate	NA	NA	NA
Total ESWL number	2 within 4 weeks	3 within 4 months	1	NA	2 within 10 days
ESWL-to-onset interval	1 week	5 months	3 months	3 months	7 months
Shock number	3200	NA	4000	1000	NA
Energy data	75 kPa	NA	NA	17 kV	NA
Renal function at last ESWL	NA	NA	Normal	Normal	Normal
Anti-GBM antibody before ESWL	NA	NA	NA	Negative	Negative

F: female; M: male; GBM: glomerular basement membrane; ANCA: anti-neutrophil cytoplasmic antibody; HLA: human leukocyte antigen; PE: plasma exchange; MP: methylprednisolone pulse; Pred: prednisone; CTX: cyclophosphamide; IgG: immunoglobulin G; C3: complement 3; ESWL: extracorporeal shock wave lithotripsy; ref: reference; Y: yes; N: no; NA: not available.

Further studies are still needed to elucidate the effect of ESWL on the initiation of anti-GBM disease. However, there has been evidence about the collagen cleaving effect of ultrasound since early 1980s when researchers tried to isolate and define the components of GBM by physical methods. Glomeruli were sequestered and sonicated to separate the GBM, which then released split products of collagen with antigenicity [35], indicating ultrasound could cause local damage of the GBM. There was also evidence of transient nephrotic-range proteinuria immediately after ESWL in a patient cohort [36] and mesangial proliferative glomerulopathy after ESWL in experimental animal models of pig [37]. Moreover, shock wave lithotripsy is utilized in pancreatic and large common bile duct stones or sialolithiasis, but no cases of anti-GBM disease have been reported in either of these conditions yet, suggesting ESWL for urinary stones may affect kidneys more directly. Therefore, ESWL could possibly damage and expose the antigen inside the GBM *via* direct damage by shock waves or secondary damage by immune-complex induced by ESWL debris [37].

Anti-GBM IgG subclass distribution is associated with disease severity [38]. The IgG1 and IgG3 dominated the IgG subclasses in patients with severe renal impairment, while IgG2 and IgG4 were associated with milder renal damage [38]. In post-ESWL anti-GBM disease patients from this study, the IgG2 and IgG4 were the dominant subclasses against α3(IV)NC1. However, these patients presented with severe renal damage and poor prognosis. The IgG subclass switching after B cell activation follows the sequence of IgG3→IgG1→IgG2→IgG4. It has been suggested that IgG4 production results from chronic or repetitive antigenic stimulation [39,40]. Moreover, low-level natural anti-GBM autoantibodies existed in healthy human sera, predominantly of IgG2 and IgG4 [41]. We speculated that ESWL might expose GBM autoantigens and induce IgG autoantibodies chronically during the ESWL-to-onset interval, allowing them to complete subclass switching. The autoantibodies may accumulate beyond a threshold to disturb the immune tolerance in healthy individuals and provoke the pathogenic autoimmunity.

Anti-GBM diseases were also reported scarcely to associate with obstructive uropathy due to urinary malignancy, neurogenic bladder, or ureteral stenosis [15–18]. The anti-GBM antibody level and renal function seemed to be parallel with treatment efficacy of hydronephrosis [18]. Intact NC1 hexamer could be detected in the serum and be secreted in the urine of healthy individuals [17,42]. Rabbits immunized with their own urinary concentrate developed anti-GBM glomerulonephritis [43]. It was suggested that urinary α3(IV)NC1 under urinary obstruction might enter the renal interstitia, dissociate under acidic pH changed by inflammatory infiltrate (such as infection) and act as an immunogen [17]. These studies provide another theory for the induction of anti-GBM disease associated with urinary stones, including patients treated by ESWL.

The limitations of our study lay in at least two points. Firstly, the number of cases is too small to draw the causal relationship between ESWL and anti-GBM disease. Moreover, we cannot rule out the possibility that individuals with anti-GBM disease or with susceptible HLA phenotypes may bear a higher risk of urinary obstructions, which lead to the production of anti-GBM antibodies. A prospective cohort study in patients undergoing ESWL might be required to further elucidate the relationship between the procedure and anti-GBM disease. Secondly, not all patients had detailed ESWL information (stone component, shock wave frequency, and energy), kidney biopsy, and HLA phenotypes for us to draw a full picture.

In summary, the anti-GBM disease could happen within weeks to months after ESWL treatment and present with similar clinical features, antigen spectrum, and prognosis to classic anti-GBM disease. IgG2 and IgG4 were the two dominant subclasses against α3(IV)NC1. Although the causal relationship between ESWL and anti-GBM disease still needs further exploration, our study here may act as a reminder for physicians that patients developing acute renal insufficiency after ESWL should lead to the suspicion of anti-GBM disease and in-time diagnosis and treatment.

## Supplementary Material

Supplemental Material

## Geolocation information

City: Beijing, Latitude: 39.9289, Longitude: 116.3883.
