# Supplementary material for: The clinical and immunological features of the post-extracorporeal shock wave lithotripsy anti-glomerular basement membrane disease
Source: Ren Fail. 2021 Jan 12;43(1):149–55. doi: 10.1080/0886022X.2020.1869042 (PMC7808748; doi:10.1080/0886022X.2020.1869042)

**Supplementary figure**

**Figure. S1. The Immunological Features of Anti-Glomerular Basement Membrane (GBM) Antibodies in Patients with Post-Extracorporeal Shock Wave Lithotripsy (ESWL) Anti-GBM Disease.** (A) Serum antibody spectrum against α1(IV)NC1–α5(IV)NC1. (B) Serum antibodies against epitope EA and EB. (C) Immunoglobulin G (IgG) subclass distribution of anti-α3(IV)NC1 antibody.


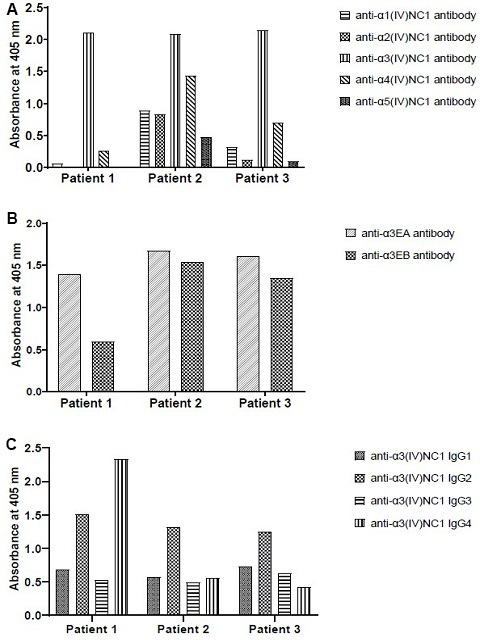

Supplement: Supplemental Material [file IRNF_A_1869042_SM8470.docx]
